# Anisotropic Resistivity Size Effect in Epitaxial Mo(001) and Mo(011) Layers

**DOI:** 10.3390/nano13060957

**Published:** 2023-03-07

**Authors:** Atharv Jog, Pengyuan Zheng, Tianji Zhou, Daniel Gall

**Affiliations:** Department of Materials Science and Engineering, Rensselaer Polytechnic Institute, 110 8th St., Troy, NY 12180, USA

**Keywords:** electron transport, interconnects, epitaxy, thin films, surface scattering

## Abstract

Mo(001) and Mo(011) layers with thickness *d* = 4–400 nm are sputter-deposited onto MgO(001) and α-Al_2_O_3_(11$\bar{2}$0) substrates and their resistivity is measured in situ and ex situ at room temperature and 77 K in order to quantify the resistivity size effect. Both Mo(001) and Mo(011) layers are epitaxial single crystals and exhibit a resistivity increase with decreasing *d* due to electron surface scattering that is well described by the classical Fuchs and Sondheimer model. Data fitting yields room temperature effective electron mean free paths λ^*^ *=* 14.4 ± 0.3 and 11.7 ± 0.3 nm, respectively, indicating an anisotropy with a smaller resistivity size effect for the Mo(011) orientation. This is attributed to a smaller average Fermi velocity component perpendicular to (011) surfaces, causing less surface scattering and a suppressed resistivity size effect. First-principles electronic structure calculations in combination with Boltzmann transport simulations predict an orientation dependent transport with a more pronounced resistivity increase for Mo(001) than Mo(011). This is in agreement with the measurements, confirming the effect of the Fermi surface shape on the thin-film resistivity. The predicted anisotropy $\lambda_{001}^{*}$/$\lambda_{011}^{*}$ = 1.57 is in reasonable agreement with 1.66 and 1.23 measured at 77 and 295 K. The overall results indicate that the resistivity size effect in Mo is relatively small, with a measured product of the bulk resistivity times the effective electron mean free path *ρ*_o_λ^*^ = (7.7 ± 0.3) and (6.2 ± 0.2) × 10^−16^ Ωm^2^ for Mo(001) and Mo(011) layers. The latter value is in excellent agreement with the first-principles-predicted *ρ*_o_*λ =* 5.99 × 10^−16^ Ωm^2^ and is 10% and 40% smaller than the reported measured *ρ*_o_*λ* for Cu and W, respectively, indicating the promise of Mo as an alternate conductor for narrow interconnects.

## 1. Introduction

The continued miniaturization of features in integrated circuits for the development of future technology nodes will drive the half pitch of back-end-of-line (BEOL) interconnects to <10 nm, introducing significant performance and reliability challenges [1,2,3,4]. Despite the advances in novel low-*k* dielectrics and liner layer scaling, the increase in the resistance-capacitance (RC) delay poses a serious threat to the damascene Cu extendibility, which has been the industrial standard for BEOL interconnects since its introduction in 2000 [5,6,7,8,9,10]. This increase in the RC delay is a direct consequence of the resistivity size effect, which is primarily attributed to electron scattering at surfaces [11,12,13,14,15,16] and grain boundaries [17,18,19,20,21] as the linewidths *w* or grain size *D* of nanoscale conductors approach and become smaller than the electron–phonon scattering mean free path, which is 39 nm for Cu [22,23,24]. The resistivity increase due to surface and grain boundary scattering has been described by the classical models developed by Fuchs and Sondheimer (FS) [25,26] and Mayadas and Shatzkes (MS) [27], respectively, which predict additive contributions to the resistivity proportional to *ρ*_o_*λ* × (1 − *p*)/*d* for surface scattering and *ρ*_o_*λ* × *R*/(1 − *R*)*D* for grain boundary scattering, where *p* and *R* are the surface scattering specularity and grain boundary reflection parameters [28]. In the limit of narrow wires, metals with a small *ρ*_o_*λ* product are promising, as a small *ρ*_o_*λ* minimizes resistivity contributions from both surface and grain boundary scattering [29,30,31,32,33].

Mo has been identified as a promising metal to replace copper and/or tungsten for the most scaled BEOL interconnects and/or middle-of-line (MOL) vertical contacts, respectively. The predicted *ρ*_o_*λ =* 5.99 × 10^−16^ Ωm^2^ for Mo is 10% and 27% smaller than the predicted values for Cu and W [34]. In addition, Mo has a two times higher cohesive energy than Cu [35], suggesting that it may not require a diffusion barrier and may possess superior electromigration resistance even in the absence of an adhesion liner layer, maximizing the volume available for metal fill in the interconnect trench [36]. The melting point of Mo (2896 K) is 22% lower than that of W (3695 K), leading to a larger expected grain size which enhances the conductance benefit of Mo compared to W for narrow vertical interconnects. Conversely, the 2.1 times higher melting point of Mo in comparison to that of Cu (1358 K) limits Mo grain growth at BEOL processing temperatures, resulting in a smaller expected average grain size for Mo than for Cu [37], which may diminish or even eliminate the conductance advantage of Mo over Cu. Other elemental metals that are considered for nanoscale conductors include Rh [38,39], Ir [40,41], and Ru [42,43,44]. They have 46%, 38%, and 15% smaller *ρ*_o_*λ* products than Mo [34], respectively, but are challenging due to cost, small earth abundance, and limited process maturity [45,46,47].

In this article, we quantify the resistivity size effect in Mo using a combination of experiments and first-principles simulations. Epitaxial Mo(001) and Mo(011) layers with thicknesses ranging from *d* = 4 to 400 nm are sputter deposited onto MgO(001) and α-Al_2_O_3_(11$\bar{2}$0) substrates, respectively. Epitaxial layers allow for quantification of electron surface scattering and its dependence on layer orientation without the confounding effects from scattering at grain boundaries. The measured resistivity of thin films of Mo(001) is larger than for Mo(011), despite that the bulk resistivity is isotropic and their resistivities are identical for thick films. This is confirmed by Boltzmann transport simulations which indicate a resistivity anisotropy, with the predicted resistivity decreasing from Mo(111) to Mo(001) to Mo(011) layers. Data fitting of *ρ* vs. *d* provides values for the effective electron mean free paths λ^*^, and *ρ*_o_λ^*^ values of (7.7 ± 0.2) and (6.2 ± 0.2) × 10^−16^ Ωm^2^ for Mo(001) and Mo(011) layers, which suggest that Mo has the potential to provide a higher conductivity than Cu and W in the limit of narrow wires.

## 2. Experimental and Simulation Methods

Epitaxial Mo(001) and Mo(011) layers were grown on single-crystal MgO(001) and α-Al_2_O_3_(11$\bar{2}$0) substrates in a three-chamber ultrahigh vacuum (UHV) magnetron sputtering system with a base pressure of 10^−9^ torr [48]. Substrates were cleaned with successive rinses in ultrasonic baths of trichloroethylene, acetone, iso-propyl alcohol, and de-ionized water for 15 min each. They were blown dry with N_2_ and mounted on a Mo holder using colloidal silver paint, introduced into the deposition system via a load-lock, and degassed in vacuum at 1000 °C for 1 h. Depositions were performed on MgO(001) and α-Al_2_O_3_(11$\bar{2}$0) substrates at *T*_s_ = 900 and 1000 °C, respectively, by applying a constant DC power to a 99.95% pure Mo target facing a continuously rotating substrate in 20 mTorr 99.999% pure Ar. The deposition time was adjusted to obtain two series of epitaxial Mo layers with *d* = 4–400 nm, as measured by X-ray reflectivity for layers with *d* < 100 nm and determined from the deposition rate for layers with *d* > 100 nm. After deposition, the samples were in situ vacuum annealed at 1000 °C for 2 h in the same UHV deposition system at < 10^−7^ torr. The described deposition temperatures and annealing procedure were optimized to maximize crystalline quality while minimizing surface roughness and dewetting for small film thicknesses. After annealing, the layers were allowed to cool to room temperature for 12 h and were subsequently transported without breaking vacuum to an analysis chamber for in situ resistivity measurements along MgO[010] and Al_2_O_3_[0001] directions using a linear four point probe operated at 1–100 mA. Samples were removed from the deposition system via a load-lock vented to atmospheric pressure using dry N_2_ and were immersed in liquid nitrogen within 2 s to limit air exposure and associated oxidation. Resistivity measurements were taken at 77 K using a similar four point probe with the probe tips completely immersed in liquid N_2_. Ex situ measurements were performed with the same setup after the samples were warmed to room temperature by blowing dry N_2_ on the Mo surface to reduce ice/water build up. The resistivity was re-measured at 77 K after 48 h of air exposure to quantify the effect of surface oxidation on electron surface scattering.

X-ray diffraction (XRD) and reflectivity analyses were performed using a Panalytical X’pert PRO MPD system with a Cu source. *ω*–*2θ* scans, *ω*-rocking curves, and *ϕ* scans were acquired using a hybrid mirror with a Ge(220) two-bounce monochromator yielding *λ* = 1.5406 Å with a beam divergence of 0.0068° and a PIXcel solid-state line detector operating in receiving mode with an active length of 0.165 mm, corresponding to a *2θ* opening of <0.04°. Rocking curves were acquired by scanning in *ω* while keeping the *2θ* value fixed to detect the desired 002 or 011 reflections. *ϕ* scans were acquired using the same diffraction equipment but with a point source and a polycapillary lens instead of a hybrid mirror on the incident beam side, providing quasi-parallel Cu K_α_ X-rays with a divergence of < 0.3°. These *ϕ* scans were obtained using a *χ* tilt of 45° and fixed *2θ* values corresponding to Mo 011 or Mo 002 reflections for 001- and 011-oriented layers, respectively. X-ray reflectivity (XRR) measurements were performed in the same system with a parabolic mirror optic yielding a parallel beam with <0.055° divergence. The measured XRR data were fitted using the PANalytical X’Pert Reflectivity software, which employs the Parratt formalism. For this purpose, the densities for Mo (a = 3.147 Å, Im$\bar{3}$m), MgO (a = 4.212 Å, Fm$\bar{3}$m), and α-Al_2_O_3_ (a = 4.758 Å, c = 12.99 Å, R$\bar{3}$c) were fixed at 10.2, 3.58, and 3.98 g/cm^3,^ respectively, while the free fitting parameters were the Mo layer thickness, the root-mean-squared (rms) surface roughness, and the rms layer–substrate interface roughness.

Atomic force microscopy (AFM) was performed using a Digital Instruments Multimode III-a AFM operating in tapping mode, employing a 4 μm thick silicon cantilever and a tip with a radius of <10 nm and a resonance frequency of 184 kHz. 500 × 500 nm^2^ micrographs were analyzed using the Gwyddion software and the surface morphology was quantified by obtaining the rms surface roughness *ω* and the lateral correlational length *ξ* determined using height–height correlation function H(*r*) analyses.

Simulations of electron transport in Mo layers were done from first principles, following an approach we previously developed and successfully applied to the cases of W [49] and Ru [42]. The electronic structure of bulk Mo with an experimental (literature value) lattice constant of 3.147 Å was determined from density functional theory calculations using the Vienna ab initio simulation package (VASP), employing periodic boundary conditions, a plane wave basis set with an energy cutoff of 225 eV, the Perdew–Burke–Ernzerhof generalized gradient approximation (GGA) exchange correlation functional, the projector augmented wave method, and a pseudo potential (Mo_pv) that includes all core electrons up to the 4s electrons such that 4p, 4d, and 5 s electrons are explicitly calculated. Self-consistent calculations using a Γ-centered 40 × 40 × 40 *k*-point grid were employed to determine the charge distribution, which was subsequently used for non-self-consistent calculations with a finer 200 × 200 × 200 *k*-point mesh. The chosen *k*-point mesh yields a computational accuracy for the ballistic conductance that is converged to ±0.4%. The Fermi surface and the *k*-vector-dependent Fermi velocity vector were determined from the calculated bands using an irregular tetrahedra method [34], yielding approximately 10^6^ triangles that define the Fermi surface and were used for subsequent numerical integration with appropriate weights, including triangle area and electron velocity components along the transport direction [34,42,49,50]. Electron transport was simulated using Boltzmann transport with a constant mean free path approximation and simultaneous integration over real and reciprocal space [42,49] of a thin film with thickness *d* and the Brillouin zone for bulk Mo. We chose to calculate the thin film vs bulk resistivity ratio *ρ*/*ρ_o_* for three layer orientations with low index surfaces (001), (011), and (111) and two in-plane transport directions for each layer orientation. This was done for 1000 *ρ*_o_*d* values that span four orders of magnitudes that are experimentally relevant; i.e., exhibit an approximately negligible to sixfold resistivity increase. These layer orientations and transport directions were chosen to quantitatively illustrate the anisotropic size effect and to directly compare the simulation results with our experimental measurements.

## 3. Results

### 3.1. Microstructural Analysis

**Figure 1:**
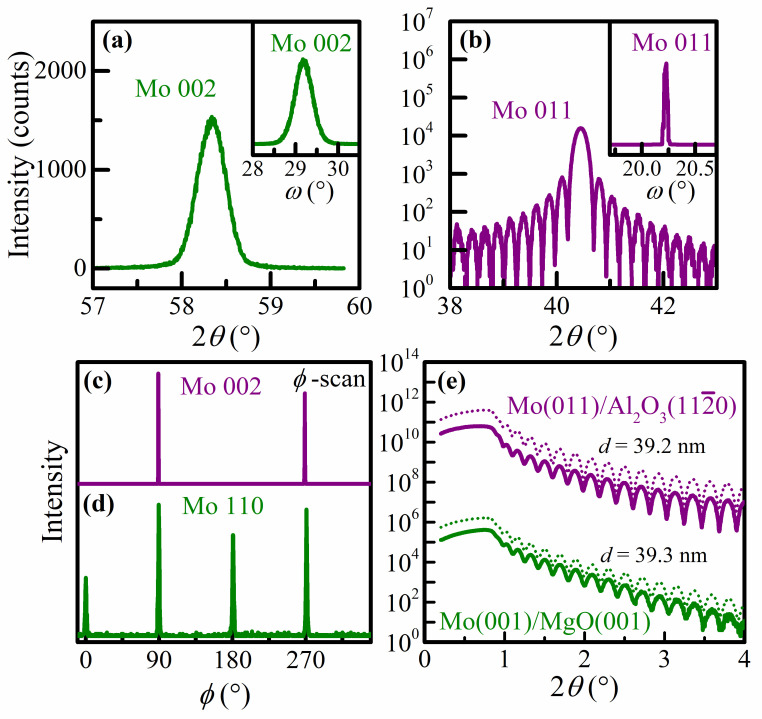
Representative (**a**,**b**) XRD *ω–*2*θ* scans, including *ω*-rocking curves from the primary Mo reflections in the insets, (**c**) Mo 002 and (**d**) Mo 110 *ϕ* scans, and (**e**) XRR curves from nominally 39 nm thick epitaxial Mo(001) (green) and Mo(011) (purple) layers grown on MgO(001) and α-Al_2_O_3_(11$\bar{2}$0) substrates, respectively.

Figure 1 shows representative X-ray diffraction and reflectivity results that demonstrate that the Mo layers grown on MgO(001) and α-Al_2_O_3_(11$\bar{2}$0) substrates are smooth epitaxial Mo(001) and Mo(011) layers. The symmetric *ω*–*2θ* scan from a 39.3 nm thick Mo/MgO(001) layer plotted in green in Figure 1a shows a peak at *2θ =* 58.33° due to the Mo 002 reflection, indicating an out-of-plane lattice constant *a* = 3.161 Å that is 0.4% larger than the reported bulk value. This indicates a slight biaxial compressive stress which is attributed to the substrate–layer misfit in lattice parameter and/or thermal expansion coefficient and causes a compressive in-plane strain of *ε* = −0.4%, as determined using a Poisson’s ratio of 0.33. This strain is expected to have a negligible (<1%) effect on transport properties, based on the assumption of a linear response for conventional metals, and is therefore not further considered in this study. The Mo 002 reflection is the only layer-peak detected over the entire measured *2θ =* 10–90° range, indicating a Mo 001 out-of-plane alignment. This is confirmed by the narrow *ω*-rocking curve for the Mo 002 peak shown in the inset of Figure 1a, which exhibits a full-width at half-maximum (FWHM) of $\Gamma_{\omega }^{002}$ = 0.49°. An XRD *ϕ* scan from the same sample is shown in Figure 1d. It is obtained with a 45° *χ-*tilt and a fixed 2*θ* = 40.41° in order to detect asymmetric Mo 110 reflections. The plot shows four peaks at 0°, 90°, 180°, and 270° that are shifted by 45° with respect to the MgO 220 reflections obtained from *ϕ* scans with 2*θ* = 62.12° (not shown), indicating a 45° rotation between the substrate and the layer. These XRD results demonstrate the epitaxial growth with Mo(001)‖MgO(001) and Mo[010]‖MgO[110], similar to what has previously been reported for the epitaxial growth of W(001)/MgO(001) [51].

Figure 1b shows an *ω*–*2θ* scan (purple) from a 39.2 nm thick Mo/Al_2_O_3_(11$\bar{2}$0) layer. The peak at *2θ* = 40.44° corresponds to the Mo 011 reflection and the well-developed Laue oscillations indicate a very high crystalline quality, smooth surfaces, and a Mo thickness of 39.9 nm, in excellent agreement with *d* = 39.2 nm from the XRR analyses. The only other layer peak that can be detected is the Mo 220 reflection at 87.18°, indicating a single 011 out-of-plane layer orientation. This is confirmed by the very narrow Mo 110 rocking curve (see inset) with a width $\Gamma_{\omega }^{011}$ = 0.03°, corresponding to an in-plane X-ray coherence length of 375 nm. The Mo 002 *ϕ* scan from the same sample as shown in Figure 1c exhibits two peaks separated by 180°, indicating twofold symmetry for the 011-oriented layer and confirming epitaxy with Mo(011)‖Al_2_O_3_(11$\bar{2}$0) and Mo[01$\bar{1}$]‖Al_2_O_3_[0001] or Mo[100]‖Al_2_O_3_[1$\bar{1}$00].

Figure 1e shows representative X-ray reflectivity curves from the same two Mo layers deposited on MgO(001) and α-Al_2_O_3_(11$\bar{2}$0) substrates with *d* = 39.3 and 39.2 nm, respectively. The measured intensities are plotted as solid green and purple curves in a logarithmic scale as a function of scattering angle *2θ* = 0–4°. The dotted curves are the result of curve fitting using the Parratt formalism and are offset by a factor of four for clarity purposes. They describe the measured characteristic Kiessig fringes well and provide values for the layer thickness of 39.3 and 39.2 nm, surface roughness σ = 0.45 and 0.42 nm, and interface roughness of 0.55 and 0.10 nm for Mo(001) and Mo(011), respectively. That is, the Mo layers are smooth on both MgO(001) and α-Al_2_O_3_(11$\bar{2}$0) substrates, and there are negligible chemical reactions at the layer–substrate interfaces. Similar XRD and XRR measurements were performed for all samples in this study (see the Supplementary Materials, Figures S1–S4), confirming epitaxy for all layers and indicating smooth surfaces and interfaces, as also summarized in Table 1. We note that for layers with *d* > 100 nm, the thickness is determined using the deposition rate and time as the spacing between XRR fringes is too small to be resolved.

**Figure 2:**
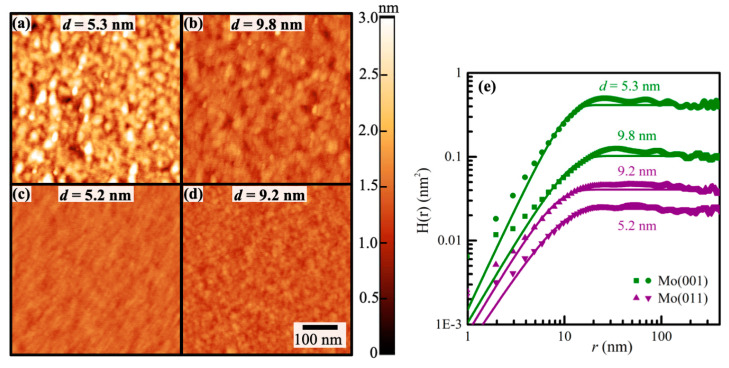
AFM micrographs (500 × 500 nm^2^) from epitaxial Mo(001)/MgO(001) layers with thickness (**a**) *d* = 5.3 nm and (**b**) 9.8 nm, and Mo(011)/Al_2_O_3_(11$\bar{2}$0) with (**c**) *d* = 5.2 nm and (**d**) 9.2 nm. The plot in (**e**) shows the height–height correlation functions H(*r*) obtained from (**a**–**d**).

Figure 2 shows typical 500 × 500 nm^2^ atomic force micrographs from four epitaxial Mo(001)/MgO(001) and Mo(011)/Al_2_O_3_(11$\bar{2}$0) layers and a plot of the corresponding measured height–height correlation functions H(*r*). The micrograph in Figure 2a is from the surface of a 5.3 nm thick Mo 001 layer which exhibits 278 ± 48 mounds, corresponding to an area number density of 1112 ± 193 μm^−2^. The average mound width *w* = 30 ± 3 nm and measured root-mean-square roughness σ = 0.45 ± 0.06 nm. The latter is in good agreement with 0.55 nm from the XRR analysis and corresponds to an average peak-to-valley surface mound height *h* = 2$\sqrt{2}$σ = 1.3 ± 0.2 nm. The micrograph in Figure 2b is also from a Mo(001) layer but with nearly twice the thickness *d* = 9.8 nm. Similar analyses yield an 86% larger mound density of 2064 ± 192 μm^−2^, a 27% smaller width *w* = 22 ± 3 nm, and a 51% smaller σ = 0.22 ± 0.05 nm, indicating a smoothening of the surface with increasing thickness that may be attributed to a transition from a nuclei-dominated to a surface island dominated surface morphology. Figure 2c,d show the corresponding surfaces of Mo(011) layers with thicknesses of 5.2 and 9.2 nm, respectively. The *d* = 5.2 nm layer exhibits 1693 ± 163 mounds/μm^2^, a small σ = 0.11 ± 0.03 nm, and *w* = 22.6 ± 1.7 nm, indicating that the Mo(011) surfaces are smoother than Mo(001), which may be partially attributed to the smoother sapphire vs magnesium oxide substrates, as well as an expected smaller activation energy for adatom surface diffusion on the more closely packed Mo(011) than Mo(001) surface. The thicker *d* = 9.2 nm Mo(011) layer has a similarly smooth surface, with σ = 0.14 ± 0.03 nm and island width *w* = 18.5 ± 2.1 nm, but a larger mound-density, with 2920 ± 132 mounds/μm^2^. This increase in island number density with increasing thickness from 5 to 10 nm is similar to what is measured for Mo(001) and is attributed to kinetic roughening in combination with nuclei coarsening, leading to a higher density of surface mounds that are sufficiently pronounced such that they are detected by AFM without increasing the overall surface roughness.

Figure 2e is a log–log plot of the height–height correlation functions H(*r*) determined from the four micrographs shown in Figure 2a–d. The plot includes as solid lines the results from data fitting using the real-space scaling function H(*r*) = 2σ^2^ [1 − exp((−r/ξ)^2α^)], following the approach described in previous work [39,52,53]. This provides values for the rms surface roughness σ (presented above), the lateral correlational length *ξ*, and the Hurst roughness exponent *α* for each sample. The measured lateral correlation lengths are consistent with the *w* values discussed above, with relatively comparable values for all four samples: *ξ* = 9.8 ± 0.6 and 10.5 ± 0.7 nm for Mo(001) with *d* = 5.3 and 9.8 nm, and *ξ* = 8.7 ± 0.8 and 7.5 ± 0.6 nm for Mo(011) with *d* = 5.2 and 9.2 nm. That is, within experimental uncertainty, *ξ* is thickness-independent for both sets of thin layers within this thickness range. This is attributed to a surface morphology that is affected by both initial nucleation as well as kinetic roughening during continued layer growth. The roughness exponent *α* = 0.87–1.2 for the four samples is close to the expected *α* = 1 for a self-affine fractal surface morphology [54]. It is slightly smaller for Mo(011) than for Mo(001), which may be attributed to the smaller kinetic barriers for adatom mobility on the more closely packed (011) surface.

### 3.2. Electron Transport

**Figure 3:**
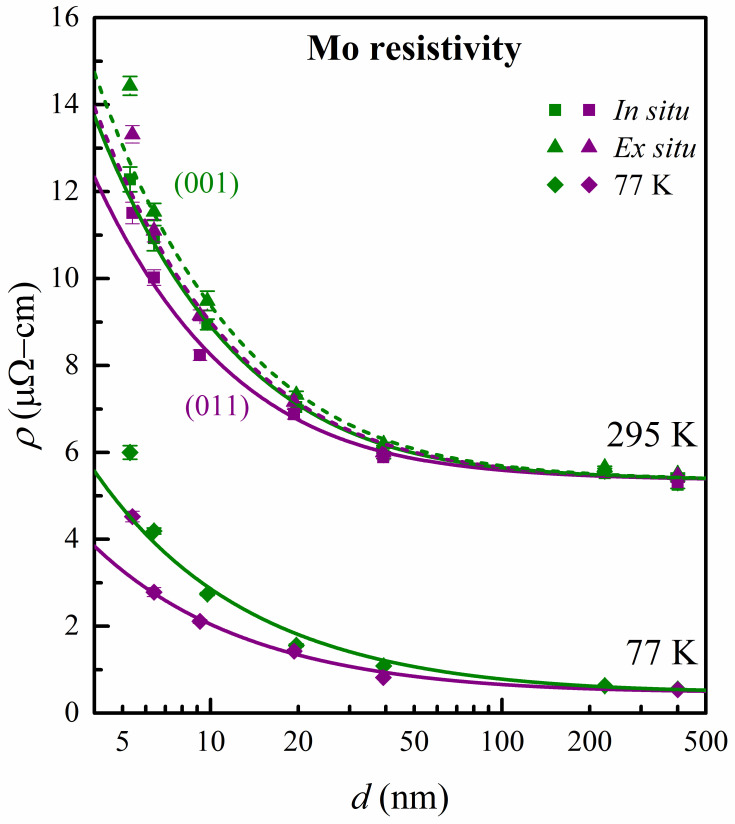
Resistivity *ρ* vs thickness *d* measured in situ (squares) and ex situ (triangles) at 295 K and immersed in liquid nitrogen at 77 K (diamonds) for epitaxial Mo(001)/MgO(001) (green) and Mo(011)/Al_2_O_3_(11$\bar{2}$0) (purple) layers, respectively.

Figure 3 is a plot of the Mo(001) and Mo(011) layer resistivity *ρ* as a function of thickness *d* measured both in situ and ex situ at 295 K and immersed in liquid nitrogen at 77 K. The resistivity increases with decreasing *d* for all datasets, as also summarized in Table 1. The resistivity increase is attributed to electron scattering at the Mo top and bottom surfaces, which becomes more pronounced with decreasing thickness. The plotted green squares for Mo(001) show an in situ resistivity *ρ* = 5.25 ± 0.08 μΩcm for the thickest layer with *d* = 400 nm. This value is identical (within experimental error) to the reported Mo bulk resistivity *ρ*_o_ = 5.34 μΩcm [55], indicating a negligible resistivity size effect for *d* = 400 nm. However, *ρ* increases with decreasing *d* to reach 12.3 ± 0.3 μΩcm at *d* = 5.3 nm. Table 1 includes an even thinner 3.9 nm thick Mo(001) layer, which is outside of the plotted range in Figure 1. This layer exhibits a partially discontinuous microstructure, as discussed below, and is, therefore, excluded from further analyses. The ex situ measured room temperature resistivity (green triangles) is consistently larger than that measured prior to air exposure. This is similar to what has previously been reported for electropositive metals, such as Nb(001) [56], Cu(001) [57,58]**,** Co(0001) [59], and Ni(001) [60], and has been attributed to diffuse electron scattering due to localized surface states [61] and charge transfer at oxygen-exposed surfaces [62]. However, the effect here for Mo is less pronounced, corresponding to a 34% change in surface scattering specularity during air exposure, while the corresponding change is 70% for Cu and more than 100% (unphysical) for Nb, Co, and Ni, as discussed in [62]. The Mo(001) resistivity at 77 K is plotted in Figure 3 using green diamonds, indicating *ρ* = 0.55 ± 0.02 μΩcm for *d* = 400 nm. This is 17% larger than the reported *ρ* = 0.47 μΩcm for bulk Mo at 77 K [55]. This deviation is attributed to electron surface scattering, which becomes significant at lower temperatures, even for a large *d* = 400 nm, due to the increased electron–phonon scattering mean free path. More specifically, the quantitative analysis described below with a 77 K mean free path of 143 ± 7 nm estimates a 14% resistivity contribution from surface scattering at *d* = 400 nm, in good agreement with the observed 17%. Correspondingly, the residual resistivity that can be attributed to electron scattering at crystalline defects in this Mo(001) sample at 77 K is 3% or 0.014 μΩcm, which is smaller than the 0.02 μΩcm experimental uncertainty and, thus, can be considered negligible. Decreasing the layer thickness increases the resistivity at 77 K, similar to the measurements at room temperature. However, the lower electron–phonon scattering at 77 K results in lower absolute values and, correspondingly, a more pronounced relative resistivity size effect. For example, the room temperature resistivity increases by 108% when the layer thickness is reduced from 400 to 6.4 nm, while the corresponding increase is 660% at 77 K.

Figure 3 also shows the resistivity of Mo(011)/Al_2_O_3_(11$\bar{2}$0) layers as purple squares and diamonds for 295 and 77 K, respectively. For a large thickness (*d* = 400 nm), the Mo(011) resistivity matches the values for Mo(001) within ~1% at both temperatures, suggesting that the lower measured crystalline quality of the Mo(001) layers has a negligible effect on the residual resistivity from electron scattering at crystalline defects. However, for *d* < 100 nm, the two datasets diverge, with the resistivity of Mo(011) being consistently smaller than that for Mo(001) layers. For example, for *d* = 6.4 nm, Mo(011) has an 8% smaller *ρ* than Mo(001) at room temperature and a 33% smaller *ρ* at 77 K.

## 4. Discussion

We now discuss electron transport in our Mo(001) and Mo(011) layers in terms of the semi-classical framework developed by Fuchs [25] and Sondheimer [26] for the resistivity in thin metallic films. The FS model quantifies the resistivity contribution due to electron surface scattering using two parameters: the electron–phonon scattering mean free path *λ* and the phenomenological surface specularity parameter *p*, which can be further divided into *p*_1_ and *p*_2_ for scattering at the top and bottom surfaces of a thin film [28]. However, the two fitting parameters are strongly correlated, and the measured thickness dependence of resistivity does not allow to uniquely determine both. Hence, to circumvent this problem, we set *p*_1_ = *p*_2_ = 0 and obtain a lower bound for the mean free path by fitting the measured *ρ* vs *d* data using the exact form of the FS model [38,40,42,59]. We refer to this quantity as the *effective* mean free path λ^*^, since it does not physically match the electron–phonon scattering length but simply defines the characteristic length scale for the resistivity size effect, therefore quantifying the resistivity increase within the FS model. The solid and dashed lines in Figure 3 are the results from curve fitting of our measured *ρ* vs *d*. This is done with the FS model by fixing *p*_1_ = *p*_2_ = 0 and the bulk resistivity *ρ*_o_ = 5.34 and 0.47 μΩcm at 295 and 77 K, respectively. Thus, the only remaining fitting parameter is λ^*^, which is independently determined for each dataset, as summarized in Table 2. This table also includes λ^*^ values from ex situ measurements at 77 K, which are obtained by immersing the samples in liquid N_2_ after room temperature air exposure for 48 h, as described in Section 2. We note that the data from the Mo(001) layer with *d* = 3.9 nm are not included in the fitting procedure because of its dramatically larger resistivity (45% larger than for *d* = 6.4 nm), which is attributed to a non-uniform microstructure at the percolation limit containing voids between thin film nuclei that have not yet coalesced; similar to what has previously been reported for, for example, Ir(001) layers with *d* < 5 nm [40]. The fitting yields effective mean free paths from the in situ data of $\lambda_{001}^{*}$ = 14.4 ± 0.3 nm and $\lambda_{011}^{*}$= 11.7 ± 0.3 nm for Mo(001) and Mo(011) layers at 295 K, respectively, indicating a 19% smaller resistivity size effect for Mo(011) than for Mo(001). Similarly, the ex situ values of 16.4 ± 0.2 and 14.8 ± 0.3 nm also indicate a smaller (by 11%) resistivity scaling for Mo(011). The corresponding effective mean free paths at 77 K, $\lambda_{001}^{*}$= 143 ± 7 nm and $\lambda_{011}^{*}$= 86 ± 3 nm, are nearly an order of magnitude larger than at 295 K. This is due to the reduced electron–phonon scattering at low temperatures which reduces the resistivity but increases the mean free path, leading to an expected constant *ρ*_o_*λ* product. Our measurements yield *ρ*_o_λ^*^ = (7.7 ± 0.2) and (6.2 ± 0.2) × 10^−16^ Ωm^2^ for Mo(001) and Mo(011) layers at room temperature and *ρ*_o_λ^*^ = (6.7 ± 0.3) and (4.0 ± 0.1) × 10^−16^ Ωm^2^ at 77 K; that is, the measured *ρ*_o_*λ* product is nearly temperature-independent, with 13% and 35% smaller values at 77 K than at 295 K. These deviations are small in comparison to the order-of-magnitude changes in *ρ*_o_ and *λ* and may be attributed to (a) a thickness-dependent electron–phonon coupling factor [63], (b) a wave vector-dependent electron–phonon scattering cross-section [64,65], and/or (c) the breakdown of the FS model in the limit of small thickness and low temperature [66]. We reiterate that this analysis does not explicitly account for electron scattering at small-angle grain boundaries, which is justified by the measured rocking curve widths ranging from 0.1 to 2.6° for Mo(001) and 0.03 to 0.14° for Mo(011), as shown in Figures S3 and S4. More specifically, considering a Burger’s vector of 2.7 Å, a 0.1° grain boundary corresponds to a line of dislocations that are separated by 155 nm. This is larger than the thickness of most studied layers, suggesting that a grain boundary effectively corresponds to a single edge dislocation. Even considering the widest rocking curves of 1.3 and 2.6° from the 9.8 and 5.3 nm thick Mo(001) layers, we find that they correspond to dislocation spacings of 11.9 and 5.9 nm, respectively which are slightly larger than the layer thicknesses, confirming that a “grain boundary” is effectively a single dislocation. Nevertheless, we note that such dislocations—which are expected to be at the substrate layer interface—may cause strain fields, resulting in additional electron scattering that is most pronounced for the thinnest layers. 

**Figure 4:**
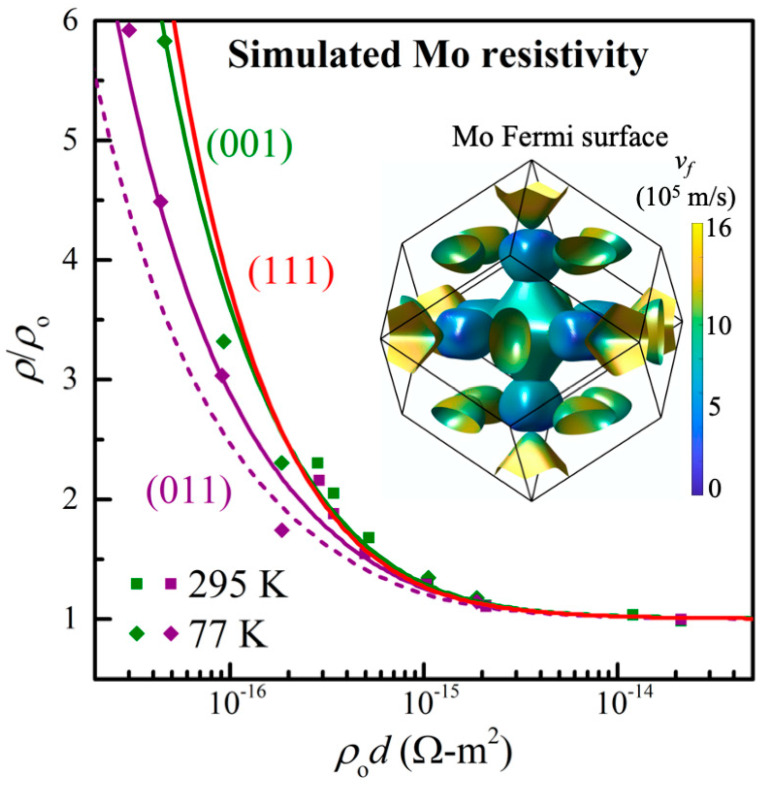
Lines: simulated Mo film resistivity *ρ*/*ρ*_o_ plotted as a function of *ρ*_o_*d* where *ρ*_o_ is the bulk resistivity, as determined from the calculated Fermi surface shown in the inset which is color coded according to the Fermi velocity *v_f_*. The green and red lines are for Mo(001) and Mo(111) layers, respectively, while the dashed and solid purple lines represent the simulated conduction along <1$\bar{1}$0> and <100> in-plane directions for Mo(011). Data points: the resistivity measured in situ at 295 K (squares) and in liquid nitrogen at 77 K (diamonds) from Figure 3.

Figure 4 summarizes the results from our transport simulations using the bulk Mo Fermi surface which is calculated from first principles and used as input data for Boltzmann transport simulations, as described in Section 2. It shows (as plotted lines) the predicted resistivity for the Mo thin films vs their thickness for three layer orientations and includes, for comparison, the measured resistivity as individual data points. For this plot, the y-axis is the thin-film resistivity *ρ* normalized by the bulk resistivity *ρ*_o_, while the x-axis is the thickness *d* multiplied by *ρ*_o_. This normalization makes the simulation results independent of the electron–phonon scattering rate so that the plotted curves are valid at all temperatures. For each layer orientation, two in-plane transport directions are simulated assuming completely diffuse surface scattering and employing a constant mean free path approximation; more specifically, Mo(001) with transport along [100] and [110], Mo(011) with transport along [100] and [01$\bar{1}$], and Mo(111) with transport along [01$\bar{1}$] and [$\bar{2}$11]. The two transport directions result in identical values (deviation < 0.1%) for Mo(001) and Mo(111), which is attributed to the fourfold and sixfold in-plane symmetry, respectively, resulting in in-plane isotropic transport. In contrast, the Mo(011) layer only has a twofold in-plane symmetry, resulting in direction-dependent electron surface scattering and, therefore, different resistivities along perpendicular [100] and [01$\bar{1}$] directions, as indicated by the solid and dashed purple lines in Figure 4, respectively. All curves converge to the same *ρ* = *ρ*_o_ for large *d*, since transport in bulk Mo is isotropic due to cubic symmetry. However, the curves diverge for small *d* with *ρ*_011_ < *ρ*_001_ < *ρ*_111_. This is due to an orientation-dependent Fermi velocity *v_f_*, as indicated by the plotted Fermi surface in the inset. The color coding indicates the *k*-dependent *v_f_* which ranges from 2 × 10^5^ to 15 × 10^5^ m/s, with an average value of 9.2 × 10^5^ m/s. The fastest electrons (yellow) are at the hole octahedron near the zone boundary along <100>, while the hole ellipsoid (green/turquoise) at the zone boundary along <110> has an approximately 20% smaller *v_f_*. Thus, electrons move faster towards Mo(001) than Mo(011) surfaces and, therefore, scatter more on surfaces of 001 oriented layers. This qualitatively explains the lower simulated resistivity of the Mo(011) layer, similar to what has previously been reported for W(011) [49]. Isotropic numerical integration over the Fermi surface yields a calculated *ρ*_o_*λ* = 5.99 × 10^−16^ Ωm^2^ [34].

The data points in Figure 4 are the measured resistivity reproduced from Figure 3 for the Mo(001) and Mo(011) oriented layers. For this plot with a normalized x-axis, each sample yields two data points where the resistivity at 77 K (diamonds) is shifted by approximately one order of magnitude to the left of the 295 K value, since *ρ*_o_ is 11 times smaller at 77 K. Overall, the simulated curves are in good agreement with the experimental data. More specifically, there is good agreement in the magnitude of the resistivity size effect obtained from experiment and simulation. The simulation also correctly predicts a smaller resistivity for Mo(011) than Mo(001) layers, as observed experimentally and indicated by the purple vs green lines and symbols. We note that measurements using a linear four-point probe results in currents that are non-parallel to the probe alignment, such that the experimental values for Mo(011) layers are effective averages of multiple transport directions parallel to (011) planes, corresponding to values somewhere between the solid and dashed purple lines.

We now interpret the transport simulation results in Figure 4 by determining effective mean free paths λ^*^ that correspond to the simulated curves. For this purpose, similar to the data analysis of the measured resistivity in Figure 3, the predicted resistivity curves in Figure 4 are fitted using the FS model with completely diffuse scattering *p*_1_ = *p*_2_ = 0 and Mo bulk resistivities *ρ*_o_ = 5.34 and 0.47 μΩcm at 295 and 77 K, respectively. This yields $\lambda_{001}^{*}$= 12.3 ± 0.1 nm for the simulated room-temperature effective mean free path for Mo(001) layers, $\lambda_{111}^{*}$ = 12.4 ± 0.1 nm for Mo(111), and $\lambda_{011}^{*}$ = 8.7 ± 0.2 and 7.0 ± 0.2 nm for Mo(011) with transport along [100] and [01$\bar{1}$], respectively, as also listed in Table 2. Thus, the resistivity size effect as quantified by the effective electron mean free path is 29–43% smaller for Mo(011) than for Mo(001) and Mo(111) layers, while the difference between the latter two layer orientations is negligible. The corresponding simulated λ^*^ values at 77 K are also listed in Table 2. They are 11.4 times larger than at 295 K, where the factor of 11.4 is the ratio between the bulk resistivities at room temperature and 77 K. We can now directly compare experimental and simulated λ^*^ values. Our experimental linear four-point probe geometry cannot measure in-plane anisotropy since it effectively measures average sheet resistances. Thus, for comparison between experiment and simulation, we determine the average $\lambda_{011}^{*}$ from the two simulated transport directions of the Mo(011) layers, yielding $\lambda_{011}^{*}$ = 7.8 ± 0.2 and 89 ± 2 nm at 295 and 77 K, respectively. We find that the simulated room temperature mean free path is 14% and 33% smaller than the experimental values for Mo(001) and Mo(011) layers, respectively. At 77 K, the simulated $\lambda_{001}^{*}$ and $\lambda_{011}^{*}$ are 2% smaller and 3% larger than those from the experiment; that is, there is good agreement between experiment and simulation, with a nearly perfect quantitative match at 77 K but a noticeable deviation at 295 K. We attribute this to the higher reliability of the experimental data at 77 K. This is because the relative magnitude of the resistivity size effect is an order of magnitude more pronounced at 77 K than at room temperature, rendering the effects due to, for example, surface morphology [67] more negligible than at 295 K. It is also illustrative to quantify the level of anisotropy between Mo(001) and Mo(011) by determining the ratio $\lambda_{001}^{*}$/$\lambda_{011}^{*}$. We find $\lambda_{001}^{*}$/$\lambda_{011}^{*}\;$= 1.23 and 1.66 from the experiments at room temperature and 77 K, respectively, while the simulated ratio is 1.57. Thus, both experiments and simulations indicate a considerably stronger resistivity size effect for Mo(001) than for Mo(011). We note that the anisotropy in the resistivity size effect, as quantified by the ratio $\lambda_{001}^{*}$/$\lambda_{011}^{*}$ of the effective electron mean free paths, is smaller for Mo than for the previously reported case of tungsten [49].

## 5. Conclusions

The anisotropic resistivity size effect in Mo is quantified using transport measurements on epitaxial layers and simulations employing the first-principles Mo electronic structure. The measured resistivity vs thickness data analyzed using the Fuchs and Sondheimer model indicate an orientation dependent bulk effective electron mean free path $\lambda_{001}^{*}$ = 14.4 ± 0.3 nm and $\lambda_{011}^{*}$= 11.7 ± 0.3 nm at 295 K for Mo(001) and Mo(011) layers, respectively, indicating a 19% smaller resistivity size effect for Mo(011) than for Mo(001). The resistivity anisotropy is attributed to an anisotropy in the Fermi velocity that results in electrons moving faster toward Mo(001) than Mo(011) surfaces, causing exacerbated electron surface scattering and a correspondingly higher resistivity in Mo(001) layers. There is good quantitative agreement between experiment and simulation regarding the effective electron mean free path, as well as regarding the magnitude of the anisotropy of the resistivity size effect. The agreement is particularly good at 77 K, which is attributed to a more pronounced resistivity size effect at low temperatures. The overall results indicate a product of the bulk resistivity times the effective electron mean free path *ρ*_o_λ^*^ = (7.7 ± 0.2) and (6.2 ± 0.2) × 10^−16^ Ωm^2^ for Mo(001) and Mo(011) layers. The latter value is in excellent agreement with *ρ*_o_*λ =* 5.99 × 10^−16^ Ωm^2^ predicted from first principles and 10% and 40% smaller than the previously reported measured *ρ*_o_*λ* for Cu and W, suggesting that Mo has the potential to outperform Cu and W in the limit of narrow wires.

## Figures and Tables

**Table 1:** Thickness *d,* surface roughness *σ*, and resistivity *ρ* measured in situ and ex situ at 295 K and immersed in liquid nitrogen at 77 K, of epitaxial Mo(001) and Mo(011) layers.

*d* (nm)	*σ* (nm)	*ρ* (µΩcm)		
		295 K		77 K
		In Situ	Ex Situ	
Mo(001)				
3.9 ± 0.2	-	15.9 ± 0.4	17.9 ± 0.5	9.17 ± 0.27
5.3 ± 0.3	0.55	12.3 ± 0.3	14.4 ± 0.2	6.04 ± 0.16
6.4 ± 0.3	0.38	10.9 ± 0.3	11.5 ± 0.2	4.18 ± 0.07
9.8 ± 0.4	0.53	8.94 ± 0.12	9.49 ± 0.22	2.74 ± 0.03
19.7 ± 0.5	0.55	7.04 ± 0.04	7.32 ± 0.08	1.56 ± 0.03
39.3 ± 0.5	0.45	5.94 ± 0.08	6.19 ± 0.04	1.08 ± 0.01
225 ± 3	-	5.52 ± 0.03	5.65 ± 0.03	0.63 ± 0.01
400 ± 5	-	5.25 ± 0.08	5.50 ± 0.02	0.55 ± 0.02
Mo(011)				
5.2 ± 0.3	0.67	11.5 ± 0.3	13.3 ± 0.2	4.52 ± 0.12
6.4 ± 0.2	-	10.0 ± 0.2	11.1 ± 0.3	2.78 ± 0.11
9.2 ± 0.4	0.32	8.24 ± 0.12	9.15 ± 0.12	2.11 ± 0.05
19.3 ± 0.5	0.43	6.88 ± 0.11	7.16 ± 0.14	1.42 ± 0.04
39.2 ± 0.7	0.42	5.88 ± 0.02	5.99 ± 0.02	0.82 ± 0.01
400 ± 5	-	5.31 ± 0.01	5.46 ± 0.02	0.54 ± 0.02

**Table 2:** Effective electron–phonon scattering mean free path λ^*^ measured from the thickness dependence of the resistivity of Mo(001) and Mo(011) layers at 295 and 77 K, and from first-principles transport simulations for Mo(001), Mo(011), and Mo(111) layers. The in-plane anisotropy for Mo(011) is indicated by two simulated transport directions [100] and [01$\bar{1}$].

λ^*^ (nm)				
Experiment	Mo(001)		Mo(011)	
	In situ	Ex situ	In situ	Ex situ
295 K	14.4 ± 0.3	16.4 ± 0.2	11.7 ± 0.3	14.8 ± 0.3
77 K	143 ± 7	153 ± 7	86 ± 3	106 ± 5
Calculations	Mo(001)	Mo(011) Mo(011)		Mo(111)
		[100]	[01$\bar{1}$]	
295 K	12.3 ± 0.1	8.7 ± 0.2	7.0 ± 0.2	12.4 ± 0.1
77 K	140 ± 2	99 ± 2	79 ± 2	140 ± 2

## Data Availability

The data presented in this study are available on request from the corresponding author.

## Supplementary Materials

The following supporting information can be downloaded at: https://www.mdpi.com/article/10.3390/nano13060957/s1, Figure S1: XRR curves from epitaxial Mo(001) layers grown on MgO(001) susbtrates. The well-developed Kiessig fringes are fit with the Parratt formalism to obtain the layer thickness as discussed in the experimental methods section. Figure S2: XRR curves from epitaxial Mo(011) layers grown on α-Al_2_O_3_(11$\bar{2}$0) substrates. The critical angle of the layer with *d* = 5.2 nm is smaller compared to the thicker layers, suggesting a microstructure at the percolation limit which is also confirmed by the AFM analysis from this layer. Figure S3: *ω*-rocking curves from the Mo(001) layers grown on MgO(001) substrates. As the layer thickness is reduced from *d* = 400 nm to 5.3 nm, the FWHM increases from 0.1° to 2.6°, in agreement with literature on sputter deposited layers. Figure S4: *ω*-rocking curves from the Mo(011) layers grown on α-Al_2_O_3_(11$\bar{2}$0) substrates. Similar to Mo(001) layers, as the layer thickness is reduced, the FWHM increases from 0.03° to 0.14°. The value of the FWHM for Mo(011) oriented layers is an order of magnitude smaller than for Mo(001), indicating superior crystallinity for Mo(011).
