# Supplementary material for: Anisotropic Resistivity Size Effect in Epitaxial Mo(001) and Mo(011) Layers
Source: Nanomaterials (Basel). 2023 Mar 7;13(6):957. doi: 10.3390/nano13060957 (PMC10052566; doi:10.3390/nano13060957)
Supplement: Supplementary file 1 [file nanomaterials-13-00957-s001.zip › nanomaterials-2220349-supplementary.pdf]

Supplementary Materials

Anisotropic resistivity size effect in epitaxial Mo(001) and Mo(011) layers

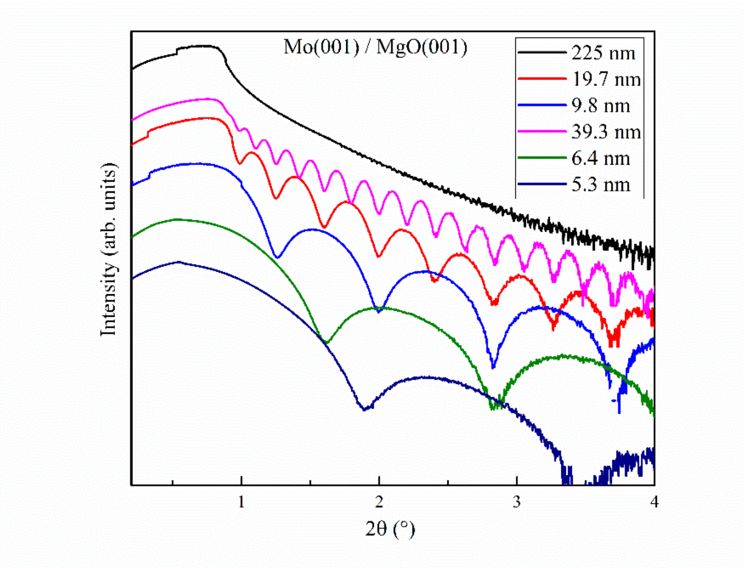

Figure S1: XRR curves from epitaxial Mo(001) layers grown on MgO(001) susbtrates. The well-developed Kiessig fringes are fit with the Parratt formalism to obtain the layer thickness as discussed in the experimental methods section.

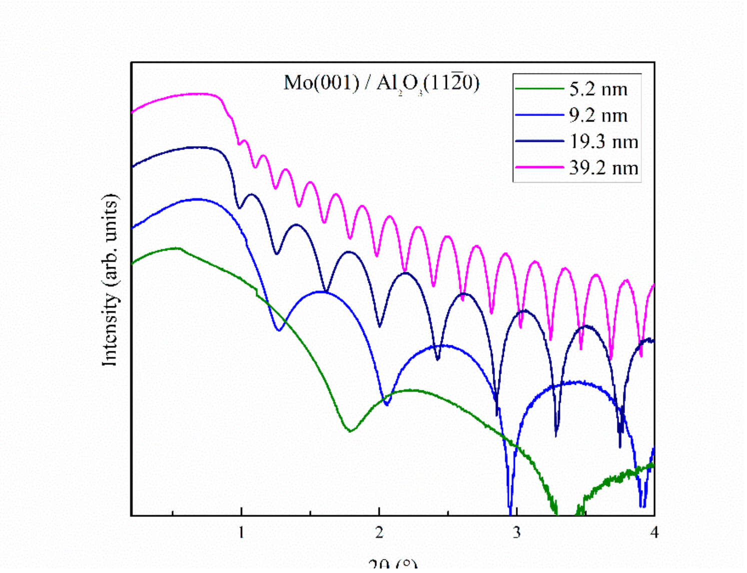

Figure S2: XRR curves from epitaxial Mo(011) layers grown on α-Al_2O_3(11$\bar{2}$0) substrates. The critical angle of the layer with d = 5.2 nm is smaller compared to the thicker layers, suggesting a microstructure at the percolation limit which is also confirmed by the AFM analysis from this layer.

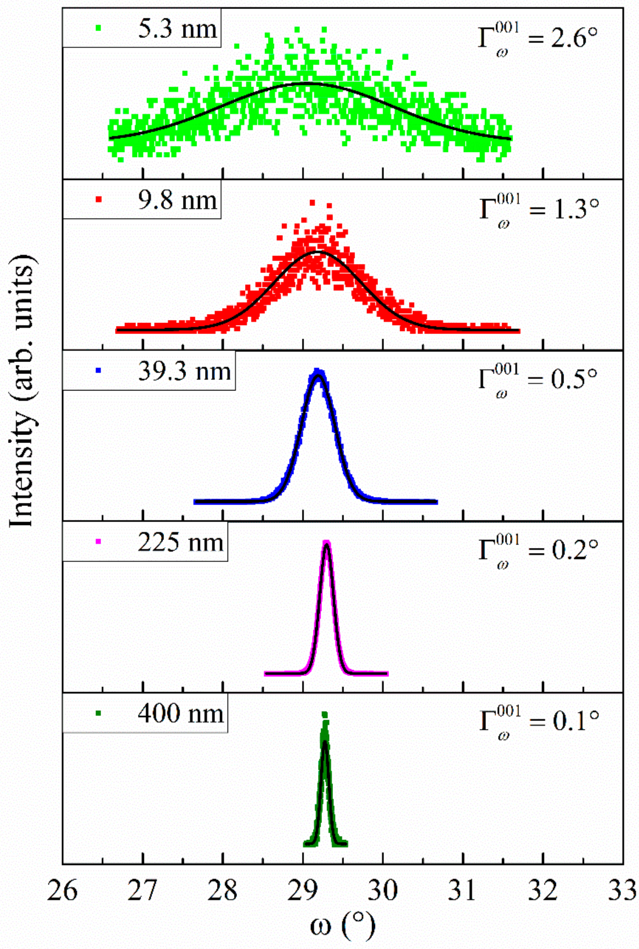

Figure S3: *ω*-rocking curves from the Mo(001) layers grown on MgO(001) substrates. As the layer thickness is reduced from *d* = 400 nm to 5.3 nm, the FWHM increases from 0.1° to 2.6°, in agreement with literature on sputter deposited layers.

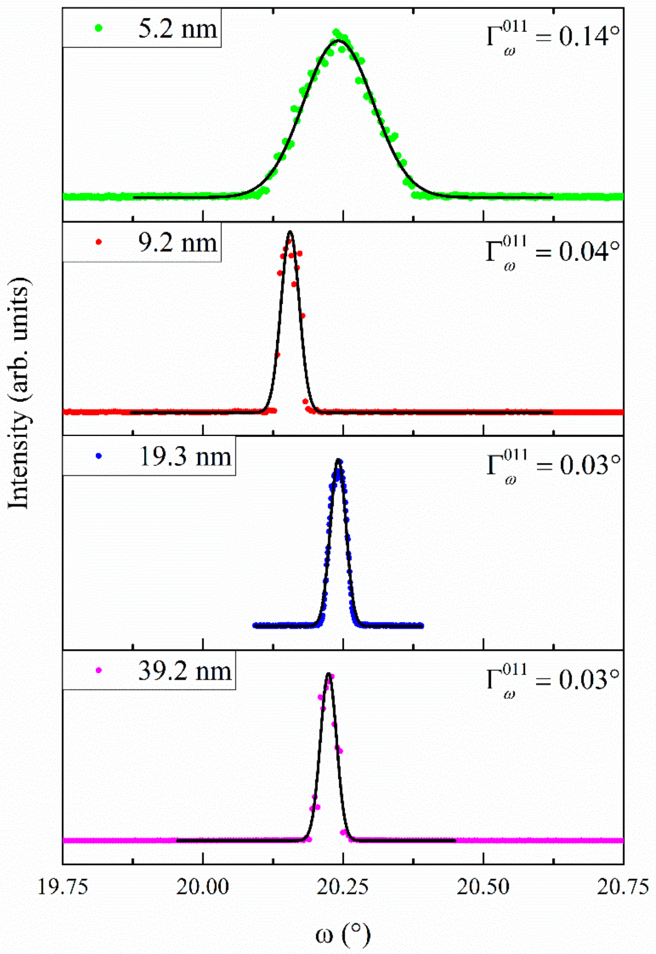

Figure S4: ω-rocking curves from the Mo(011) layers grown on α-$Al_2O_3(11\bar{2}0)$ substrates. Similar to Mo(001) layers, as the layer thickness is reduced, the FWHM increases from 0.03° to 0.14°. The value of the FWHM for Mo(011) oriented layers is an order of magnitude smaller than for Mo(001), indicating superior crystallinity for Mo(011).
